# Antibiotic tolerance

**DOI:** 10.1371/journal.ppat.1008892

**Published:** 2020-10-15

**Authors:** Lars F. Westblade, Jeff Errington, Tobias Dörr

**Affiliations:** 1 Department of Pathology and Laboratory Medicine, Weill Cornell Medicine, New York, New York, United States of America; 2 Division of Infectious Diseases, Department of Medicine, Weill Cornell Medicine, New York, New York, United States of America; 3 The Centre for Bacterial Cell Biology, Biosciences Institute, Newcastle University, Newcastle upon Tyne, United Kingdom; 4 Weill Institute for Cell and Molecular Biology and Department of Microbiology, Cornell University, Ithaca, New York, United States of America; 5 Department of Microbiology, Cornell University, Ithaca, New York, United States of America; 6 Cornell Institute of Host-Pathogen Interactions and Disease, Cornell University, Ithaca, New York, United States of America; Tufts Univ School of Medicine, UNITED STATES

## Introduction

**Fig 1:**
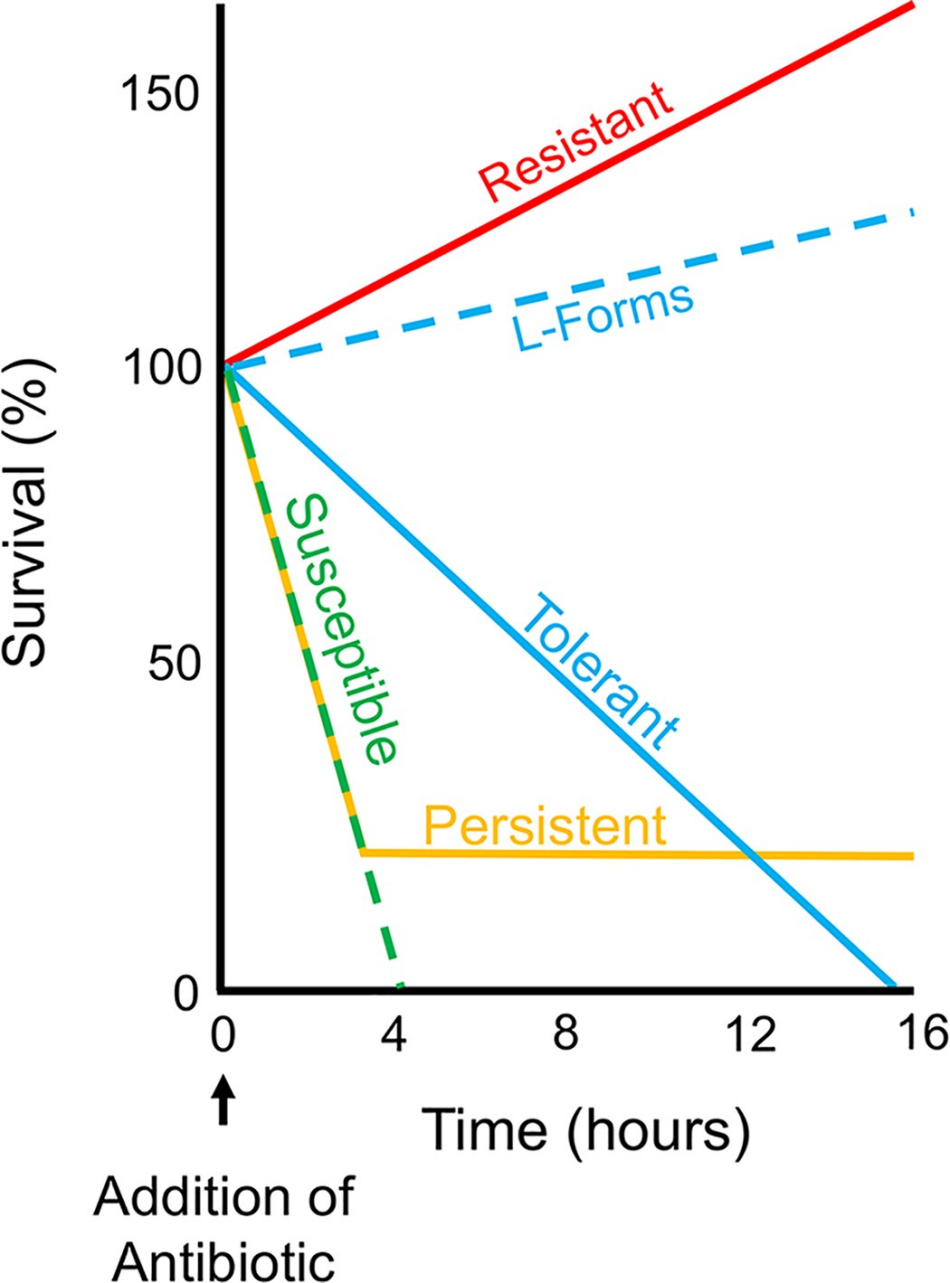
Graphical depiction of the phenomenological definitions of susceptible, resistant, tolerant, and persistent (i.e., extreme tolerance). A hypothetical time course antibiotic kill experiment is displayed graphically. A bactericidal antibiotic (e.g., a β-lactam) is added at 0 hours followed by measurements of cell viability over time. A fully susceptible population is rapidly killed (dashed, green-colored line), while a resistant population continues to grow in the presence of the antibiotic (red-colored line). Susceptible, L-form tolerant cells continue to actively replicate (dashed, blue-colored line); albeit at a reduced rate compared to resistant cells. In contrast, a susceptible, tolerant spheroplast population (blue-colored line) does not replicate and is ultimately killed but displays prolonged viability compared to susceptible cells. Finally, in the setting of extreme tolerance (so-called “persistence”), the majority of the population is rapidly killed, but a small subpopulation remains viable for an extended period (orange-colored line).

Antibiotics have enabled the most important advances in healthcare and their clinical implementation ranks as one of the most significant medical achievements. Understandably, the often observed and increasingly prevalent failure to treat bacterial infections with antibiotics has emerged as a major public health concern. Treatment failure can be caused by 2 separate, distinct phenomena: antibiotic resistance (simplistically defined as a bacterium’s ability to grow in the presence of antibiotic concentrations that are presumed inhibitory in vivo based upon in vitro antibiotic susceptibility testing data [1]), and antibiotic tolerance (including persistence [2]), frequently defined as an antibiotic susceptible microorganism’s ability to survive extended periods of exposure to bactericidal antibiotics (**Fig 1**). In addition, the phenomenon of heteroresistance (the appearance of a small subpopulation of spontaneously resistant cells) has come into focus [3]. While a plethora of excellent work is available describing overt resistance (including heteroresistance) [3,4], antibiotic tolerance has remained understudied, and therefore, its contribution to antibiotic treatment failure remains poorly understood. Compounding the issue is the lack of available methodology to accurately assess antibiotic tolerance clinically. Herein, we provide a brief overview of the mechanisms and clinical implications of antibiotic tolerance with the hope of inspiring the scientific community to address this fundamental subject.

## Antibiotic stress response and tolerance—Why do some cells die and others don’t?

Antibiotics induce various forms of cell damage. For example, fluoroquinolones convert topoisomerases into endonucleases causing DNA damage; β-lactams and other inhibitors of cell wall (peptidoglycan) synthesis destroy the essential bacterial cell wall; polymyxins damage membranes; and aminoglycosides corrupt the translation machinery [4]. An important mechanism for surviving antibiotic exposure is for bacteria to respond by upregulating damage repair functions. As such, the survival of a subset of cells—tolerance—is often mediated by specialized stress responses. Below we present examples of how various stress responses enable bacteria to tolerate antibiotic exposure.

The SOS DNA damage response senses and repairs damage induced by fluoroquinolones and is critical for survival after exposure to these agents [5], while cell envelope stress-sensing systems promote bacterial survival in the presence of cell wall and cell envelope–targeting antibiotics [6]. The two-component VxrAB system, which is required for β-lactam tolerance in *Vibrio cholerae*, is induced by cell wall damage and upregulates peptidoglycan synthesis in response [7]. Additionally, both the Rcs phosphorelay and the envelope stress responsive alternative sigma factor RpoE have been implicated in β-lactam tolerance in gram-negative bacteria [8,9], and while their contributions are poorly understood, they suggest a role for general outer membrane/cell envelope integrity in tolerating β-lactams. The heat shock response promotes survival in the presence of aminoglycosides via upregulation of chaperones and proteases that reduce misfolded protein-induced stress [10], and responses (e.g., superoxide dismutases and catalases) that mitigate damage caused by reactive oxygen species (ROS) have been shown to be effective against a variety of antibiotic classes [11]. Antibiotic tolerance strategies mediated by stress responses are likely operating in a race against time: cells must respond to a potentially lethal insult in sufficient time to mount a response and repair the antibiotic-induced damage. Therefore, one would predict the magnitude of stochastic stress response induction before antibiotic exposure might be predictive of the probability to survive exposure, which could potentially explain population heterogeneity in survival phenotypes. Perhaps consistent with this idea, the ability to survive antibiotic exposure via elaboration of stress responses can depend on the antibiotic concentration [5].

Importantly, tolerance has been shown to be a stepping stone toward the development of frank resistance [12–14]. It is feasible that stress responses play a major role in this phenomenon, as the ability of damaged cells to repair and recover can coincide with an increase in mutation rate [15] favoring the emergence of overtly resistant mutant strains. Understanding stress response-mediated modulation of antibiotic activity in the absence of frank resistance is an understudied field that could aid in the development of novel treatment options.

## Tolerating cell wall–acting antibiotics through cell wall depletion

An important aspect of the tolerance phenomenon lies in the role of environmental conditions. The most striking example of this is the role of osmolarity on survival of cells treated with cell wall–damaging agents, particularly β-lactams. Under hypotonic conditions, peptidoglycan provides bacteria with the ability to withstand the higher turgor pressure that would otherwise result in explosive lysis but in iso-osmotic conditions, such as those present in tissue fluids and urine; the requirement for a cell wall is reduced, and the mechanical damage inflicted by cell wall–active antibiotics is mitigated. Indeed, many bacteria can adapt, to some extent, to loss of their cell wall. These peptidoglycan-deficient cells are derived, often reversibly, from wild-type bacterial cells and have been given a plethora of names, including L-forms, spheroplasts, and S-cells [16], although what all these have in common is the ability to circumvent the essentiality of the cell wall, despite their different behaviors in the presence of antibiotics (**Fig 1**). Part of the reason for the varied nomenclature is that the ability of these cells to survive and grow in the presence of antibiotics depends greatly on the organism, culture medium and conditions, and how the switch to the cell wall–deficient state is induced.

Detailed work on *Bacillus subtilis* has shown that the switch to a fully viable growing L-form state requires at least 3 steps. First, escape of the membrane-bound protoplast from the enveloping peptidoglycan sheath, usually dependent on external hydrolytic enzymes or β-lactam–induced autolysis [17]. Second, protection from osmotic lysis, usually requiring an isotonic medium containing a high concentration of divalent cations [18]. Third, protection from damage by endogenous ROS that arise, at least in part, due to the metabolic imbalances that occur when the flux of precursors to peptidoglycan synthesis is diverted [19]. Similar factors are probably involved in the formation of cell wall–deficient forms in many other bacteria, both gram-positive and gram-negative [18].

Spheroplasts are formed by many gram-negative bacterial species after exposure to inhibitors of cell wall synthesis [20–22]. Similar to L-forms, the activity of cell wall lytic enzymes is required for spheroplast formation [20]. Recovery from the spheroplast state requires cell envelope stress responses, cell wall synthesis functions, and likely, a reduction in the formation of ROS [23]. The key difference between spheroplasts and L-forms is that the former do not divide. We postulate that, in reality, the distinction between growing L-forms and nongrowing spheroplasts is purely related to whether the growth conditions, and degree of damage from ROS and other endogenous factors, permit the peptidoglycan-deficient cells to divide. The same may apply to the recently described S-cells [24], which again may be peptidoglycan-deficient cells that are incapable of division but can nevertheless remain in that state before reverting to the normal walled form.

## Extreme tolerance: Dormancy as a means to wait out antibiotic exposure

Persisters are defined as a small subpopulation of extremely tolerant, presumably dormant, cells observed in many bacterial populations. Persisters remain viable after a susceptible majority population is rapidly killed by a bactericidal antibiotic (**Fig 1**) [2,25]. As such, persistence has been classified as “heterotolerance” [25], i.e., a subclass of tolerance. At least 2 types of persisters exist: those that are induced by antibiotic exposure and other environmental factors, and those that form spontaneously [2]. A diverse range of physiological states can result in persister phenotypes, including the overproduction of toxins and other proteins [26]. Thus, persisters are not defined by the mechanistic details of their formation but only by their survival phenotype (i.e., their ability to remain viable for extended time periods in the presence of bactericidal antibiotics). Conceptually, persisters can be considered the most extreme form of tolerance as persistent cells are in a dormant, or near-dormant, physiological state. Indeed, reduction of intracellular ATP levels (a proxy of decreased metabolic activity) can correlate with persister formation [27]. By slowing down their metabolism, microorganisms are able to reduce the ability of cellular processes corrupted by antibiotic action to cause major, lethal damage. For example, β-lactam antibiotics typically only efficiently kill growing cells with ongoing cell wall synthesis [28]. Therefore, a cell that enters a nongrowing state is, in theory, protected from the lethal action of these antibiotics, and this is indeed observed in model organisms [29,30]. However, even the absence of growth does not always render cells β-lactam tolerant: nongrowing stationary phase cells of the etiologic agent of Lyme disease, *Borrelia burgdorferi*, remain susceptible to cell wall–acting antibiotics [31], suggesting at least some degree of cell wall turnover in the absence of growth.

What appears to be dormancy may in fact represent a gradient of metabolic activity rather than complete metabolic shutdown, providing varying degrees of protection depending on the antibiotic class [32]. Highly tolerant *Escherichia coli* cells that persist in the presence of the β-lactam ampicillin (“ampicillin persisters”) during exponential phase show no signs of cellular damage following antibiotic exposure [30]. In contrast, the fluoroquinolone ciprofloxacin extensively damages, but does not kill, *E*. *coli* persister cells [33]. Thus, some persisters, rather than being completely dormant, may have an exceptional ability to repair antibiotic-induced damage, perhaps favored by an already reduced metabolism that decreases damage caused by the corrupted target.

## Clinical relevance of tolerance—Does it matter?

Despite an obvious logical connection between tolerance and antibiotic treatment outcomes, studies addressing this issue remain scarce, and the clinical significance of tolerance is unclear. It is often challenging to distinguish the effects of tolerance from other factors: growth in a niche that limits antibiotic penetration, proximity to the producer of an enzyme that inactivates antibiotics, or niche-specific expression of resistance factors. While work in animal models has suggested an in vivo role for tolerance in treatment outcomes, clinical evidence is scarce [34,35].

The most notorious antibiotic tolerant bacterium is *Mycobacterium tuberculosis*, where treatment of disease caused by susceptible isolates, as determined by in vitro antibiotic susceptibility testing, requires several months of antibiotic therapy. *M*. *tuberculosis*’ high tolerance is likely a consequence of its extremely slow growth rate in the lung [36]. Antibiotic tolerance also appears to determine infection outcomes in other bacterial species. In a study of 30 patients (≥65 years of age) with recurring urinary tract infections (UTIs), cell wall–deficient bacteria were observed in fresh urine collected from 29 of the 30 patients [37]. Notably, *E*. *coli* isolates from the patients converted to a cell wall–less form in the presence of a cell wall–acting antibiotic and transitioned to the walled state upon removal of the antibiotic. Thus, switching between walled (susceptible) and cell wall–deficient (tolerant) forms may contribute to recurrence in UTIs.

While additional evidence is needed to firmly establish the significance of antibiotic tolerance on treatment outcomes, clinical isolates of significant bacterial pathogens exhibit high-level tolerance against many antibiotics [21,22,38], and several bacteria have been shown to evolve higher levels of tolerance/persistence during antibiotic therapy [14,39,40]. Lastly, conditions likely to be encountered in the human host can induce the formation of antibiotic tolerant cells (that retain the ability to produce virulence factors) [41,42]. Therefore, and especially given that tolerance can serve as a stepping stone toward the development of frank resistance [12–14,43], it is highly likely that tolerance is a major contributor to antibiotic treatment failure.

## The future of tolerance research

Future research should thoroughly address the clinical significance of antibiotic tolerance. To do so, 2 important hurdles must be overcome. First, assays analogous to standard antibiotic susceptibility testing methods such as disk/gradient diffusion or broth microdilution that detect antibiotic tolerance in the clinical laboratory must be implemented. Such assays must be facile to perform and the resultant data readily interpreted by end users. The tolerance disk test (TDtest) and an associated variant [44,45], which are based upon disk diffusion, could be viable options for detecting and quantifying tolerance, although the time to result is increased compared to standard antibiotic susceptibility testing methods which could impact clinical outcomes. Second, clinical studies focused on assessing the significance of antibiotic tolerance (as determined by a tolerance assay) on treatment outcomes are of utmost importance. These objectives, in addition to deeper mechanistic studies of antibiotic tolerance and potential avenues for therapeutic interventions, should have high priority in the ongoing fight to boost the efficacy of our dwindling antibiotic armamentarium.
